# Supplementary material for: Predicting visual function by interpreting a neuronal wiring diagram
Source: Nature. 2024 Oct 2;634(8032):113–23. doi: 10.1038/s41586-024-07953-5 (PMC11446822; doi:10.1038/s41586-024-07953-5)
Supplement: Supplementary file 6 — CRF and ERF predictions for individual TmY4 and TmY9 cells. Analogous to Supplementary Data 3, but for TmY target types. Shown are the top four monosynaptic pathways, the strongest pathway passing through each of the top ten intermediary types (ranking from Extended Data Fig. 7), and the trisynaptic pathway Tm1–TmY–Dm3–TmY (see the section entitled Prediction of spatial normalization). [file 41586_2024_7953_MOESM6_ESM.zip › DataS4/TmY9q/720575940627749931.pdf]

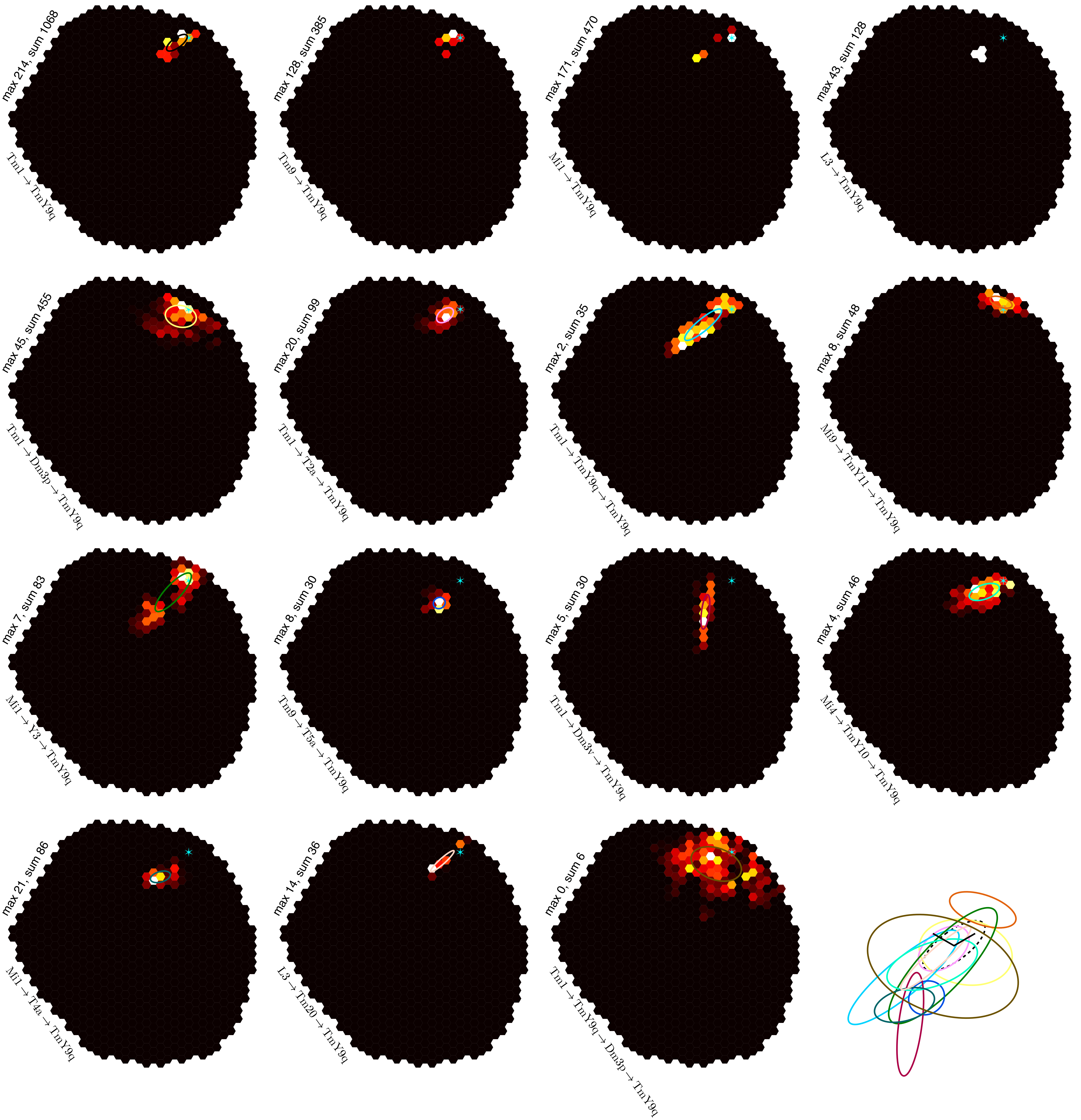
max 214, sum 1068
Tm1 → TmY9q
max 128, sum 385
Tm9 → TmY9q
max 171, sum 470
Mi1 → TmY9q
max 43, sum 128
L3 → TmY9q
max 45, sum 455
Tm1 → Dm3p → TmY9q
max 20, sum 99
Tm1 → T2a → TmY9q
max 2, sum 35
Tm1 → TmY9q → TmY9q
max 8, sum 48
Mi9 → TmY11 → TmY9q
max 7, sum 83
Mi1 → Y3 → TmY9q
max 8, sum 30
Tm9 → T5a → TmY9q
max 5, sum 30
Tm1 → Dm3v → TmY9q
max 4, sum 46
Mi4 → TmY10 → TmY9q
max 21, sum 86
Mi1 → T4a → TmY9q
max 14, sum 36
L3 → Tm20 → TmY9q
max 0, sum 6
Tm1 → TmY9q → Dm3p → TmY9q
